# Cocoa Polyphenols and Gut Microbiota Interplay: Bioavailability, Prebiotic Effect, and Impact on Human Health

**DOI:** 10.3390/nu12071908

**Published:** 2020-06-27

**Authors:** Vincenzo Sorrenti, Sawan Ali, Laura Mancin, Sergio Davinelli, Antonio Paoli, Giovanni Scapagnini

**Affiliations:** 1 Department of Pharmaceutical & Pharmacological Sciences, University of Padova, 35131 Padova, Italy; 2 Department of Biomedical Sciences, University of Padova, 35131 Padova, Italy; laura.mancin@phd.unipd.it (L.M.); Antonio.paoli@unipd.it (A.P.); 3 Department of Medicine and Health Sciences “V. Tiberio”, University of Molise, Via de Sanctis s.n.c, 86100 Campobasso, Italy; s.ali@studenti.unimol.it (S.A.); Sergio.davinelli@unimol.it (S.D.); Giovanni.scapagnini@unimol.it (G.S.); 4 Human Inspired Technology Research Center, University of Padova, 35131 Padova, Italy

**Keywords:** cocoa, polyphenols, flavanols, bioavailability, gut microbiota, human health

## Abstract

Cocoa and its products are rich sources of polyphenols such as flavanols. These compounds exert antioxidant and anti-inflammatory activities, accountable for cocoa health-promoting effects. However, cocoa polyphenols are poorly absorbed in the intestine, and most of them cannot reach the systemic circulation in their natural forms. Instead, their secondary bioactive metabolites are bioavailable, enter the circulation, reach the target organs, and exhibit their activities. In fact, once reaching the intestine, cocoa polyphenols interact bidirectionally with the gut microbiota. These compounds can modulate the composition of the gut microbiota exerting prebiotic mechanisms. They enhance the growth of beneficial gut bacteria, such as *Lactobacillus* and *Bifidobacterium*, while reducing the number of pathogenic ones, such as *Clostridium perfringens*. On the other hand, bioactive cocoa metabolites can enhance gut health, displaying anti-inflammatory activities, positively affecting immunity, and reducing the risk of various diseases. This review aims to summarize the available knowledge of the bidirectional interaction between cocoa polyphenols and gut microbiota with their various health outcomes.

## 1. Introduction

Cocoa and its derivatives are abundantly consumed worldwide due to their pleasant taste and numerous functional effects. To date, several studies have demonstrated that consumption of cocoa and its products decreases the risk of cardiovascular diseases and metabolic disorders, positively affects the immune and nervous systems, prevents the risk of cancer, and shows systemic and intestinal anti-inflammatory activities [1]. Cocoa phytochemical constituents, particularly polyphenols, are strongly associated with health-promoting benefits. Cocoa polyphenols have effective antioxidant and anti-inflammatory properties, interacting with various relevant pathways associated with several health advantages. For instance, flavanol-rich cocoa and cocoa derivatives increase nitric oxide (NO) synthesis, augment flow-mediated dilation (FMD) and enhance microcirculation [2,3], induce vasodilation and reduce blood pressure [4], attenuate platelet aggregation and improve endothelial and vascular function [1,3,4,5,6,7,8].

Furthermore, cocoa flavanols regulate lipid synthesis and degradation and glucose homeostasis and reduce the risk of obesity-induced metabolic disorders due to an increase in the expression of Peroxisome Proliferator-Activated Receptors, specifically PPAR-γ [2,5,9,10]. Cocoa flavanols also exert neuroprotective properties and improve cognition, as they can cross the blood–brain barrier (BBB) into the brain [11]. Their neuroprotective actions can be direct by enhancing MAPK, ERK, PI3 signaling pathways related to an increase in brain-derived neurotrophic factor (BDNF) expression, which promotes neurogenesis and the growth of synaptic connection and neuronal viability. Indirectly, they can exert their action by improving endothelial function, increasing blood flow, and glucose supply to the brain [12,13]. In elderly and memory-declined subjects, cocoa flavanols have been shown to induce a positive modulation of cognitive performance and improvements in several cognitive domains [14].

In recent years, the scientific community has begun to study cocoa mechanisms of action, by considering the interaction between cocoa polyphenols and the gastrointestinal microbiota, and the subsequent health outcomes.

The gut microbiota is a complex and diverse community of trillions of microorganisms, having substantial effects on physiology, metabolism, and immunity of the host [15]. Among the many known factors, dietary patterns have a significant impact on the composition and function of the gut microbiota. The influence of diet on microbiota modulation is evident from breastfeeding to the introduction of solid foods throughout life [16,17]. Dietary polyphenols are also known to interact bidirectionally with the gut microbiota and selectively promote or inhibit microbial growth and proliferation [18]. The diversity and specificity of these microorganisms are essential for polyphenol metabolism to produce secondary bioactive metabolites that interact with human biochemical pathways [19].

Likewise, cocoa polyphenols appear to modulate microbial diversity by promoting the proliferation of some bacteria and inhibiting the potentially pathogenic ones, exerting prebiotic mechanisms [20,21,22]. Deciphering this bidirectional interaction allows us to understand the mechanism of action of cocoa polyphenols and to customize its dosages and type for human health.

Therefore, this review aims to summarize and examine the metabolism and bioavailability of cocoa polyphenols and their bidirectional interactions with gut microbiota, focusing on cocoa’s prebiotic properties, from the available experimental and clinical studies.

## 2. Cocoa Composition and Processing

Botanically, cocoa (*Theobroma cacao*) is a typical evergreen tree belonging to the Sterculiaceae family, originally from tropics of Central and South America and then grown in the other parts of the world [23]. The plant has three main cultivars, with different phytochemical content and sensory properties: Criollo, the first cultivated group, Forastero, the hardier and the most prevalent group, and Trinitario, a cross-breed of both Criollo and Forastero groups [23,24]. Every year, millions of tons of cocoa are produced. The seeds of the fruits, cocoa beans, are then processed into cocoa powder, butter, and liquor [25]. Cocoa beans contain water, lipids, proteins, fibers, and many biologically active compounds [23]. In total, around 380 compounds have been identified in cocoa [1]. Among them, polyphenols and xanthine alkaloids are predominant, comprising nearly 14–20% of the bean weight [24]. Cocoa processing leads to a vast decrease in phytochemical concentration and changes their proportion [1]. In plants, phenolic compounds are involved in antioxidant activity, protection from environmental stressors such as UV radiation, microbial and fungal infection, and accordingly aiding the plant development [26].

**Figure 1:**
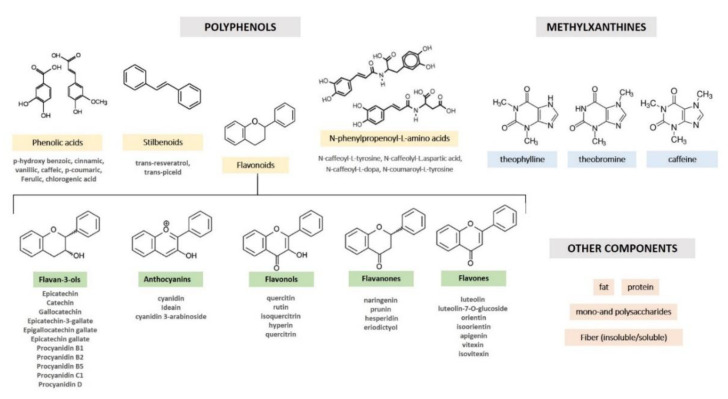
Nomenclature and chemical structures of cocoa phytocomplex.

Cocoa is especially abundant in flavanols, accounting for around 60% in non-fermented cocoa beans [27]. Cocoa flavanol comprises monomeric forms, (+)-catechin and (−)-epicatechin, and their oligomeric and polymeric forms, procyanidins. The types cocoa flavanols entail (−)–epicatechin, being the most abundant, (+)−catechin, procyanidin B1, and B2, and other flavanols at trace level, such as epigallocatechin, epigallocatechin-3-gallate, procyanidin B2-O-gallate, procyanidin B2-3,3-di-O-gallate, procyanidin B3, procyanidin B4, procyanidin B4-O-gallate, procyanidin C1, and procyanidin D (Table 1) [28]. Other minor polyphenolic compounds include flavones (luteolin, luteolin-7-O-glucoside, orientin, isoorientin, apigenin, vitexin, and isovitexin), flavanones (naringenin, prunin, hesperidin, and eriodictyol), flavonols (quercetin, quercetin-3-O-arabinoside, isoquercitrin, and hyperoside), anthocyanidins (cyanidin, 3-α-l-arabinosidyl cyanidin, 3-β-d-arabinosidyl cyanidin, 3-β-D-galactosidyl cyanidin), and certain phenolic acids (Figure 1) [29]. 

Cocoa polyphenolic profile and concentration varies depending on the type of cultivar, quality of crop and cultivation site, geographic area, and the climate. Its content also considerably varies among different cocoa products, influenced by processing and manufacturing steps [23,24]. To estimate their variations, researchers examined the polyphenol profiles and antioxidant properties of different cocoa liquor samples from six different geographic places (Madagascar, Mexico, Ecuador, Venezuela, Sao Tome, and Ghana). They observed that similar types of polyphenol compounds are present, but their proportion and antioxidant activities are different. Among them, (−)-epicatechin ranged from 0.16 to 0.59 mg/g defatted cocoa liquorsDCL and (+)-catechin varied between 0.02 and 0.42 mg/g DCL. (−)-Gallocatechin was not found in Ghana and Venezuela cocoa liquors but in other samples ranged from 0.06 to 0.40 mg/g DCL. Likewise, (−)-epigallocatechin content estimated to vary between 0.15 and 0.42 mg/g DCL, although it was not detected in Mexico and Venezuela samples. Overall, total polyphenolic contents were in the following order: Madagascar > Mexico > Ecuador > Venezuela > Sao Tome > Ghana. They also observed that higher polyphenolic content corresponds to higher antioxidant activity, indicating the contributing role of polyphenols in the antioxidant property of cocoa [24].

The production of cocoa products from cocoa beans takes several steps, and its chemistry changes by each step of the processing. It starts with drying and a five-to-seven day of fermentation, which is carried out in specific containers with a temperature of 45–50 Celsius [25]. The beans are then broken to separate the nib from its shell and subsequently sterilized. This is followed by alkalization process by an alkali solution of potassium or sodium carbonate at a temperature of 80–100 Celsius [30]. Subsequently, the alkalized product is roasted and ground to reduce the nibs to liquor or pressed to separate the fat content from the powder and eventually to produce cocoa powder and butter [31]. These final products are widely used to make chocolate, candies, and other cocoa derivatives.

Fermentation is an essential step in cocoa bean processing, during which cocoa polyphenols are oxidized enzymatically by polyphenol oxidase or non-enzymatically; then, they are polymerized and bind with proteins, resulting in high-molecular compounds (tannins) with reduced solubility and astringency. Eventually, these alterations contribute to the final product color and flavor [32,33]. During this step, epicatechins and soluble polyphenols reduce by 10–20% due to oxidation and cocoa sweating. Anthocyanins, which gradually vanish during fermentation, are used as an index for determining the fermentation degree since they hydrolyze into anthocyanidins and polymerize with catechins to form tannins. Likewise, procyanidin level and fermentation degree are negatively associated, with their levels being reported to decrease three to five-fold once cocoa is fermented [34].

On the other hand, during fermentation, the total polyphenol amount is positively influenced by the presence of lactic acid bacteria, acetic acid bacteria, and yeasts. In contrast, molds and aerobic spores have the opposite effect. Notably, it is described that a more extended fermentation period leads to the growth of fungi and the production of aerobic spores, which in turn have a negative impact on polyphenol quantity and quality [32]. 

Considerable loss of cocoa polyphenols takes place during drying, alkalization, and roasting as well [24]. During drying, the polyphenol content is substantially reduced by enzymatic browning. For instance, a 2-day drying of cocoa beans causes a 50% reduction in epicatechin [34]. Depending on temperature and timing, the roasting step also results in reduced astringent taste due to the degradation of the polyphenols [31]. In a study, researchers observed that (−)-epicatechin and (+)-catechin enormously decreased once cocoa fermented, while the enantiomer, (−)-catechin, is formed. In the same manner, high-level roasting reduced (−)-epicatechin and (+)-catechin and increased (−)-catechin and alkalization led to a continuous decrease of the monomeric flavanols and a lesser loss of (−)-catechin [35]. 

In a study to determine the changes during cocoa processing, researchers noticed that roasting and alkalization of cocoa nibs had a major effect in altering the polyphenol content. Roasting resulted in a 27% loss of (−)-epicatechin, from 2.23 mg/g to 1.63 mg/g, while the (+)-catechin content increased from 0.28 mg/g to 0.33 mg/g, which was suggested to be due to its epimerization into (−)-catechin. Procyanidin B1 decreased by 57%, from 0.28 mg/g to 0.12 mg/g and procyanidin B2 declined by 30%, from 0.63 mg/g to 0.44 mg/g. Grounding of the roasted cocoa beans into cocoa liquor led to decrease in (−)-epicatechin by 25%. Subsequently, preparing cocoa baking chocolate further declined the (−)-epicatechin, (+)-catechin, procyanidin B1 and procyanidin B2 into 0.52 mg/g, 0.24 mg/g, 0.04 mg/g and 0.16 mg/g, respectively. Furthermore, alkalizing and drying the roasted and grounded cocoa beans reduced (−)-epicatechin procyanidin B1 by 64% and 60% to 0.49 mg/g and 0.05 mg/g, respectively. Procyanidin B2 decreased by 80% while catechin increased by 27% [36].

Therefore, depending on the type of cocoa processing, the polyphenol content varies considerably, and this must be considered for cocoa health outcomes (Table 2). Furthermore, information on these profile changes helps to find the optimal conditions to reduce the loss of the polyphenol content during processing.

## 3. Cocoa Polyphenols Metabolism and Bioavailability

Polyphenols represent a large heterogeneous group of compounds that in plants are generally found as aglycones, esters, glycosides, and polymers. Once ingested, they are recognized by the human body as xenobiotics, and their absorption rate depends on the complexity of their chemical structures more than their concentration. Polyphenols generally have low bioaccessibility and bioavailability, and most of them cannot be absorbed in their natural forms. The metabolic fate of polyphenols is influenced by multiple factors: interpersonal differences in polyphenol metabolisms (genetic polymorphisms of metabolic enzymes, efflux pumps, and transporters, etc.), bidirectional interaction with the intestinal microbiota, and synergies or antagonism with other xenobiotics or nutrients [46]. All of these mechanisms modulate the polyphenol rate of absorption, distribution, metabolism, and excretion. 

**Figure 2:**
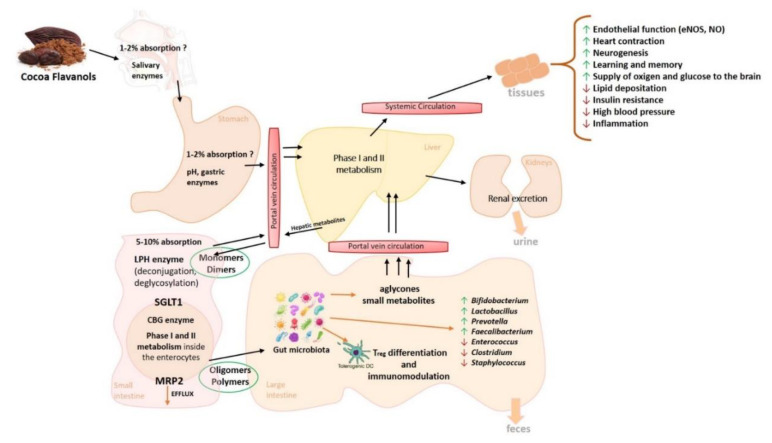
Bioavailability and properties of cocoa polyphenols.

Instead, the type of metabolism strongly affects the fate of polyphenols in the human body. In particular, once ingested, polyphenols need to be extensively modified by hydrolyzation, conjugation, and microbial degradation into their secondary metabolites that are bioactive and bioavailable [47]. Colonic microbial biotransformation of polyphenols seems to be the most effective way to produce small bioavailable secondary metabolitesable to enter the circulation, reach the target organs, and exhibit their activities [47,48]. It is estimated that only 5–10% of polyphenols are absorbed in the small intestine; the remaining 90–95% reach the colon, where they undergo fermentation processes by the intestinal microbiota into their metabolites with various physiological consequences that influence intestinal ecology and human health (Figure 2) [49,50].

Cocoa polyphenols’ metabolism begins in the oral cavity. In saliva, flavonoid glucosides are converted into aglycones and subsequently into other bioactive compounds that are absorbable by oral epithelium. Once in the stomach, oligomeric polyphenols are transformed into their monomeric units [51]. Cocoa polyphenol metabolites absorbed in the small intestine reach the liver via the portal circulation, and they undergo phase I and II biotransformation reactions, producing new metabolites. Phase I enteric and hepatic biotransformation involves oxidation, reduction, and hydrolysis of the metabolites. The following Phase II involves their conjugation that leads to the formation of [46,52] water-soluble conjugated metabolites (glucuronide, sulfate, and methyl derivatives), rapidly released into the systemic circulation for subsequent release in the target organs [53,54]. Therefore, these various metabolites reach their plasma peak nearly 2 h following oral intake [27,49]. Several sulfates, glucuronide, and methyl metabolites of epicatechin have been identified in the plasma and urine after ingestion of 100 g of dark chocolate [55]. 

The remaining glycosides, esters, and polymers require hydroxylation by small intestinal enzymes, or they reach the colon and undergo extensive metabolism by the intestinal microbiota [46,56]. As a result, two main metabolites, phenyl-γ-valerolactone and phenylvaleric acid, can be found in the urine after 5–10 h from oral intake of cocoa or its products, and they represent two main cocoa metabolites to be potentially used as a marker of cocoa polyphenol biotransformation [57].

The biotransformation of (−)–epicatechin and its procyanidin B2 dimer by human fecal microbiota has been assessed in vitro. No more than 10% of procyanidin B2 was converted to epicatechin by cleavage of the inter-flavanic bond. Some phenolic acid catabolites have been shown for both substrates. The dominant catabolites detected were 5-(3’-hydroxy phenyl) valeric (9), 3–(3’-hydroxyphenyl) propionic (10) acid and phenylacetic acid [58].

Cocoa epicatechin availability is strongly influenced by the intestinal microbiota. In a recent randomized, blinded crossover study on healthy men and women, the bioavailability and metabolites of polyphenols contained in two soluble cocoa products were evaluated: a standard and a flavanol-rich product [59]. Among the multiple metabolites, some phase II derivatives of epicatechin were consistent with the first absorption of cocoa flavanols in the small intestine. However, the predominant group of identified metabolites corresponded to those formed in the colon, such as hydroxyphenyl-γ-valerolatones and phenylvaleric acid. In the urine 5- (hydroxyphenyl)–γ-valerolactone 30-sulfate has been detected at high concentrations and could be used as a biomarker for the intake of flavanol-rich foods [59]. 

Recently, to increase cocoa polyphenol bioavailability, different cocoa-based interventions have been developed [60,61]. In food, microencapsulation of cocoa polyphenols in cocoa and hazelnut creams or the addition of bioactive components such as dietary fiber and other polyphenols in new soluble cocoa products are described [61]. In most studies, however, it has been shown and partially confirmed that flavanols and their circulating metabolites mediate the health benefits of cocoa consumption underlying the significant importance of cocoa flavanol metabolism by the intestinal microbiota.

In oral cavity, flavonoids glucosides are converted into aglycones and subsequently absorbed. In the stomach, some polyphenols undergo a first reduction into monomeric units [51]. Then, in the small intestine, a small amount of polyphenols are absorbed, mainly after de-conjugation reactions such as de-glycosylation. Aglycones can be absorbed in the small intestine, whereas glycosides, esters, and polymers typically require a first hydroxylation by the small intestinal enzymes or they reach colon microflora [46,56] and undergo microbial degradation. In addition, polyphenols absorbed in the upper part of the gastrointestinal tract—metabolized by the liver and excreted in the bile or directly extruded by the efflux pumps of the small intestine enterocytes—reach the colon and experience microbial fermentation or fecal elimination [52].

Polyphenol glycosides are hydrolyzed by two main enzymatic mechanisms in the small intestine: Lactase phenylin hydrolase (LPH) and cytosolic β-glucosidase (CBG). Once absorbed in the small intestine, residual polyphenolic compounds undergo Phase I enteric and hepatic biotransformation (oxidation, reduction, and hydrolysis) and, subsequently, Phase II reactions (conjugation) [46,52]. These transformations generate water-soluble conjugated metabolites (glucuronide, sulfate, and methyl derivatives), are released into the systemic circulation, and reach the target organs [53,54]. In the large intestine, microbial enzymes influence the 90–95% unabsorbed polyphenols and sequentially produce bioactive metabolites with various physiological consequences that influence intestinal ecology and affect human health [62,63]. Legend: MRP2 (Multidrug Resistance Associated Protein 2), SGLT1 (sodium-dependent glucose transporter 1); LPH (lactase phenylin hydrolase); CBG (cytosolic β-glucosidase).

## 4. Cocoa Polyphenols and Gut Microbiota

There is ongoing research to reveal how cocoa polyphenols shape gut microbiota and what effects the microorganisms, in turn, have on polyphenol metabolism and human health. Recent studies argue that unmodified dietary polyphenols, as well as part of the aglyconic metabolites, may alter the microflora community by exhibiting prebiotic effects and selective antimicrobial action against pathogenic intestinal microbes [47,64,65].

Out of the many gut microbial species, *Escherichia coli*, *Bifidobacterium* sp., *Lactobacillus* sp., *Bacteroides* sp., and *Eubacterium* sp. are shown to be mostly responsible for the cocoa’s polyphenol metabolism (Figure 2) [18]. Colonic microflora transform cocoa polyphenols into small bioactive compounds, which have the ability to influence the intestinal ecology and to be better absorbed into the systemic circulation instead of aglycones produced in the upper part of the gastrointestinal tract [47]. Polyphenol supplementation enhances the growth of beneficial gut bacteria, such as *Lactobacillus* and *Bifidobacterium*, while reduces the number of *Clostridium* pathogenic species, for instance *Clostridium perfringens* [66]. 

The two-way interaction between cocoa polyphenols and intestinal microbiota with the health benefits related to their prebiotic properties is discussed in the following paragraphs and summarized in Table 3.

### 4.1. In Vitro and In Vivo Studies

Several in vitro and in vivo studies underlying cocoa polyphenol interactions with the gut microbiota have been conducted. Flavanols positively influence the growth rate of *Bifidobacterium* and *Lactobacillus* but reduce the number of *Clostridium histolyticum*, as demonstrated by Tzounis et al., when they incubated fecal bacteria with flavanol. They also noted that flavanol does not have any considerable effect on the growth of *Clostridium coccoides* and *Eubacterium rectale*. Further, over the incubation period, a decrease of flavanol forms was observed, indicating their degradation by fecal microbiota [67]. 

Fogliano et al. examined the in vitro digestion of water-insoluble cocoa fraction (WICF) and its fermentation by gut enzymes and human colonic models [68]. WICF exerted prebiotic activities, by forming, in the last section of the gastrointestinal tract, a high concentration of 3-hydroxyphenylpropionic acid (3-HPP) as a result of flavanol bacterial biotransformation into phenolic acids [68].

The addition of cocoa powder in dairy products stimulates the growth of probiotic bacteria, without increasing the risk of cross-contamination with enteric pathogens. As shown in an in vitro study, where the addition of 3% cocoa powder significantly stimulated the abundance of beneficial bacteria, including *Lactobacillus* and other bacteria residing in milk. In contrast, the growth of three main food pathogens, *Escherichia coli* enterohemorrhagic O157: H7 (EHEC), *Salmonella typhimurium,* and *Listeria monocytogenes* were significantly inhibited within 9 h of ingestion. Furthermore, cocoa powder reduced the adhesion and invasion of these pathogens in a dose-dependent manner [69].

Cocoa-enriched food considerably decreases the fecal proportion of *Bacteroides*, *Clostridium,* and *Staphylococcus* genera in rodents and positively modulate the intestinal immune system, by altering colon toll-like receptors (TRR) pattern [70]. Furthermore, cocoa husks exert its positive effect on gut microflora by improving the relative proportion of different gut microbial phyla, as observed by fecal analysis of cocoa-husk fed pig models, in which a decrease in the abundance of Firmicutes (*Lactobacillus*−*Enterococcus* group and *Clostridium histolyticum*) and an increase in Bacteroidetes (*Prevotella* group and *Faecalibacterium prausnitzii*) population was reported. The production of short-chain fatty acids (SCFA) by the gut microbial community showed positive health effects, leading to a reduced risk of various intestinal inflammatory diseases [71]. In the same way, cocoa powder alters gut microbial metabolites and increases the growth of *Lactobacillus* and *Bifidobacterium* groups in pigs [72].

In an animal model of diabetes, Zucker diabetic fatty (ZDF) rats, cocoa-rich diet modifies diabetes-induced gut microbial changes to that of lean rats. It increases the relative abundance of acetate-producing bacteria, *Blautia* particularly, while decreases lactate-producing bacteria, mainly *Enterococcus* and *Lactobacillus* genera. In ZDF rat colon, cocoa elevates the levels of the tight junction protein Zonula occludens-1 (ZO-1) and mucin glycoprotein and reduces the expression of pro-inflammatory cytokines such as tumor necrosis factor-α (TNF-α), interleukin-6 (IL-6), and monocyte chemoattractant protein 1 (MCP-1). Furthermore, the biochemical parameters related to glucose homeostasis and intestinal integrity are improved [73].

Similar effects were reported in orally sensitized animal models by Camps-Bossacoma et al., in which cocoa consumption altered microbiota pattern by inducing the number of Tenericutes and Cyanobacteria phyla while reducing Firmicutes and Proteobacteria phyla. Although a cocoa diet did not influence the amount of Bacteroidetes phylum, it increased some families from this phylum, for example, *Prevotella* genus and *Bacteroides uniformis.* These results could be associated with cocoa’s polyphenol content since they are increased in humans who consume red wine polyphenols daily, and *Prevotella* is abundant in people consuming plant-rich diets [74]. In addition, cocoa-fed animals had an increase in the relative abundance of *Lactobacillus reuteri*, known for its immunomodulatory properties on Th1/Th2 regulation. This could explain the attenuation of Th2 responses induced by cocoa consumption [75] and the regulation of immunological tolerance through stimulation of Treg cells [74,75].

Cocoa polyphenols are not exclusively responsible for gut microflora alterations; other phytochemicals, such as theobromine, influence and modify gut microbiota, as well. As have been examined in rodents, cocoa containing diet that is rich in theobromine and fiber has more modulatory effects on gut microbiota than polyphenols [76]. Similar results were seen in a two-week animal-model study, in which the intake of cocoa or cocoa’s theobromine alone, lowered the amount of *E. coli* in fecal samples compared to a standard diet, indicating the role of cocoa components in reducing the abundance of gram-negative bacteria. In the same manner, the ingestion of theobromine, but not total cocoa, led to a decrease in *Bifidobacterium* spp., *Streptococcus* spp., *Clostridium histolyticum,* and *Clostridium perfingens*. These studies suggest that the different outcomes might be a consequence of a counteracting or enhancing association between different cocoa components, namely, polyphenols and fibers, with theobromine [77].

### 4.2. Clinical Studies

In humans, it seems challenging to determine the exact effects of cocoa polyphenols on the gut. Firstly, because of the low number of clinical trials, to date. Secondly, due to the complex relationship between gut microbes and polyphenols that are not only influenced by each other but also by other factors, such as interpersonal differences in gut microflora, polyphenol concentration, food matrix, and the presence of other nutrients [49,78].

The effect of food matrix has been observed in a twenty-one-participant trial, where the matrix in which the cocoa powder was dissolved, significantly increased or decreased urine concentration of different phenolic acids that are a part of gut microbial degradation pathways of flavanols. Colonic microbial metabolism of cocoa’s flavanol seemed to be affected by the consumption of cocoa with milk, but not water [79], indicating that cocoa polyphenols lead to different biological effects in different conditions.

Among the gut microorganisms, *Bifidobacterium* and *Lactobacillus* are examples of bacteria that promote human wellbeing [80]. These bacteria, along with some others, metabolize cocoa polyphenols and their growth would be mutually affected by them. The bifidogenic effect, enhancement of Bifidobacteria growth, by cocoa polyphenols is primarily due to their ability to create a redox environment in the gut that positively selects those bacteria. In a healthy gut, these polyphenols largely lead to beneficiary consequences, and in an unhealthy gut, they cause an improvement of the microbiota composition [81].

Consumption of cocoa flavanol-based food increases the number of Lactobacilli and Bifidobacteria in humans. Subsequently, the presence of these bacteria influences immunological tolerance by promoting the differentiation of the Treg cells that produce Il-10. It is suggested that cocoa flavanols might contribute as prebiotics to maintain this immunomodulation process by interfering with the intestinal microbiota [65]. Additionally, the alteration of the total number of fecal bifidobacteria and lactobacilli is associated with decreased concentration of C-reactive protein (CRP). Then, reduced CRP is linked to lower cardiovascular risk issues [18].

The aforementioned effects of cocoa flavanols were reported by Tzounis et al., in which a four-week consumption of flavonol-enriched cocoa drinks significantly enhanced the growth of *Bifidobacterium* and *Lactobacillus* groups but decreased the number of pathogenic bacteria, for example, *Clostridium histolyticum*. Similarly, the development of *Clostridium perfringens* was prevented, which is a bacterium with a possible role in the development of colon cancer and contributes to the risk of intestinal inflammatory disease. The findings also showed that the alteration of gut microbiota is linked to a variation in the triglycerides concentration in the blood [18].

A recent clinical trial investigated the prebiotic potential of dark chocolate derived from Trinitario cocoa beans, with or without lycopene incorporated in its matrix, in healthy individuals with moderate obesity. A 10 g bar of dark chocolate was quantified to contain 1.5 mg of catechins, 6.6 mg of epicatechins, 1.9 mg of dimer-B2, 7.5 mg of caffeine, 75 mg of theobromine, 75 µg of phenylethylamine, 55 µg of serotonin, and ≤ 0.1 µg of resveratrol. The results showed that dark chocolate consumption, leads to a decrease in Bacteroidetes abundance but an increase in the relative abundance of *Lactobacillus*; a reduction of liver associated blood markers of oxidative damage and inflammation and a dose-dependent change in the gut microbiota profile, blood, liver metabolism, skeletal muscle, and skin parameters were noted [82].

## 5. Conclusions and Future Perspectives

Cocoa and its products have been demonstrated to have many health benefits, primarily due to their polyphenol content. The polyphenolic profile varies between different cocoa cultivars, growing conditions, geographic areas, and processing steps. The bioavailability and health outcomes of cocoa polyphenols depend on their chemical structures and concentration, the host-related factors, and their interaction with other nutrients and the food matrix. Likewise, there might be a counteracting or enhancing association between different cocoa’s components, such as between polyphenols and fibers with theobromine. Many cocoa polyphenols reach the colon, where they are degraded into their smaller metabolites by gut microbiota. Together with their metabolites, these compounds shape the microbial population. Several experimental and clinical studies have reported the prebiotic effects of cocoa, in which they enhance the growth of beneficial bacteria such as *Lactobacillus* and *Bifidobacterium* but reduce the number of harmful ones, namely certain species of *Clostridium* genus. Finally, cocoa consumption health-promoting effects have been observed in numerous studies, primarily through their antioxidant and anti-inflammatory activities. However, the mechanisms are yet to be fully characterized. Therefore, more studies are necessary to understand cocoa polyphenols and the gut bacteria interplay fully and to determine conclusive evidence about the impacts on human health.

## Figures and Tables

**Table 1:** Phytochemicals’ concentrations in cocoa powder.

Phytochemical		Mg/g of Fresh Weight	Reference
**Flavanols**	(−)-Epicatechin	0.63- 3.3	[37,38]
	(+)-Catechin	0.61–2.02	[36,38]
	Procyanidin B1 (dimer)	1.12	[38]
	Procyanidin B2 (dimer)	0.13- 2.62	[37,38]
	Procyanidin C1 (trimer)	0.05- 0.36	[37]
	Cinnamtannin A2 (tetramer)	0.31–0.56	[37]
	Galactopyranosyl-ent-(−)-epicatechin (2alpha-->7, 4alpha->8)-(−)-epicatechin (Gal-EC-EC)	0.02–0.07	[37]
**Flavonols**	Quercetin	0.0032–0.2	[39,40]
	Isoquercitrin	0.03334–0.04664	[39]
	Quercetin-3-Arabinoside	0.05258–0.09212	[39]
	Quercetin 3-O-glucuronide	0.00938–0.01512	[39]
**Phenolic acids**			
**Hydroxybenzoic acids**	Benzoic acid	0.00017–0.00097	[41]
**Hydroxycinnamic acids**	Caffeoyl aspartate	0.37	[38]
**Hydroxybenzaldehydes**	Vanillin	0.00014–0.00232	[41]
**Methylxanthines**	Theobromine	4.11–27.69	[42,43]
	Caffeine	0.946–6.58	[42,43]
**Others**	Catechol	0.0012	[44]
	Pyrogallol	0.0018	[44]

**Table 2:** (+)-catechin and (−)-epicatechin content of cocoa bean and its derivatives.

Source	Quantity (mg/g)		Reference
	(+)-Catechin	(−)-Epicatechin	
Cocoa bean	0.28	2.23	[33]
Roasted cocoa bean	0.33	1.63	[33]
Alkalised cocoa nibs	0.26	0.58	[33]
Alkalised cocoa powder	0.60	0.88	[33]
Dried alkalised cocoa nibs	0.33	0.49	[33]
Cocoa liquor	0.34	1.22	[33]
Cocoa liquor (Venezuela)	0.14	0.74	[37]
Cocoa liquor (Brazil)	0.63	5.77	[37]
Baking chocolate	0.24	0.52	[33]
Dark chocolate (Albert Heijn)	0.1324	0.3274	[45]
Dark chocolate (Verkade)	0.1075	0.5025	[45]
Milk chocolate	0.05–0.12	0.18–0.24	[36]
Milk chocolate (Albert Heijn)	0.0383	0.1249	[45]
Milk chocolate (Verkade)	0.0269	0.1261	[45]
Baking chips	0.26–0.50	0.66–1.07	[36]
Chocolate candy bar	0.0217	0.0625	[45]

**Table 3:** The effect of cocoa and its derivatives on the growth of gut bacteria from in vitro, in vivo and clinical studies.

	Type of Cocoa	Gut Bacteria			Growth Rate	Health Outcome	Reference
		Phylum	Genus	Species			
**In vitro studies**	Water-insoluble cocoa fraction (WICF) of alkali-treated commercial cocoa powder.	Actinobacteria	*Bifidobacterium*		Increased	Exertion of antioxidant action by insoluble polyphenols	[68]
		Firmicutes	*Lactobacillus*				
	Commercial, non-alkali treated, defatted cocoa powder	Firmicutes	*Lactobacillus*	*casei*	Increased	- Diminished adherence and invasion ability of pathogenic gut bacteria	[69]
			*Lactobacillus*	*rhamnosus*			
			*Lactobacillus*	*plantarum*			
			*Lactobacillus*	*acidophilus*			
			*Bacillus*	*subtilis*			
			*Enterococcus*	*faecalis*			
			*Streptococcus*	*thermophilus*			
		Proteobacteria	*Escherichia*	*coli* O157:H7	Decreased		
			*Salmonella*	*Typhimurium*			
		Firmicutes	*Listeria*	*monocytogenes*			
In vivo studies	Natural Forastero cocoa powder	Bacteroidetes	*Bacteroides*		Decreased	- Lower body weight gain - Downregulated colon TLR2 and TLR7 and TLR9 - Reduced intestinal IgA concentration - Prevented age-related percentage increase of IgA-coating bacteria	[70]
		Firmicutes	*Staphylococcus*				
			*Clostridium*				
	Cocoa husks	Bacteroidetes	*Bacteroides−Prevotella*		Increased	- Increased fibrous fractions of the diet, mainly ADL	[71]
			*Faecalibacterium*	*prausnitzii*			
		Firmicutes	*Lactobacillus−Enterococcus*		Decreased		
			*Clostridium*	*histolyticum*			
	Flavanol-enriched cocoa powder (Acticoa)	Firmicutes	*Lactobacillus*		Increased	- Decreased TNF-a, TLR2, TLR4 and TLR9 expression of intestinal tissues	[72]
		Actinobacteria	*Bifidobacterium*				
	Natural Forastero cocoa powder	Firmicutes	*Blautia*	*Hansenii wexleare* others	Increased	- Reduced body weight - Decreased glycaemia, insulinaemia and HbA1c - Lower insulin resistance state and enhanced beta-cell function - Improved glucose tolerance - Greater crypt depth - Promoted colonocyte proliferation and apoptosis - Elevated mucin glycoprotein Increased Zonula occludens-1 (ZO-1) proteins - Partially dropped level of TNFα, IL-6, MCP-1 and CD45 lin the colonic mucosa	[73]
		Bacteroidetes	*Flavobacterium*				
		Deferribacteres					
		Cyanobacteries					
		Firmicutes	*Enterococcus*		Decreased		
			*Lactobacillus*				
		Bacteroidetes	*Parabacteroides*	*Goldsteinii distasonis* others			
		Proteobacteria	*Sutterella*				
	Cocoa powder	Firmicutes	*Lactobacillus*	*reuteri*	Increased	- Lower intestinal IgA concentration	[74]
			*Anaerostipes*				
		Bacteroidetes	*Prevotella*				
			*Bacteroides*	*uniformis*			
		Tenericutes					
		Cyanobacteria					
		Firmicutes	*Ruminococcus*	*flavefaciens*	Decreased		
			*Holdemania*				
		Proteobacteria					
	Natural Forastero cocoa powder	Firmicutes	*Butyrivibrio*		Increased	- Lower body weight gain - Higher SCFA (acetic, propionic, butyric and formic acids) - Smaller percentages of fecal IgA-coated bacteria	[77]
		Bacteroidetes	*Prevotella*				
		Cyanobacteria					
		Firmicutes	*Anaerotruncus*		Decreased		
		Bacteroidetes	*Bacteroides*	*acidifaciens*			
**Human studies**	Dairy-based cocoa-beverage mixes (standardized flavanol content)	Actinobacteria	*Bifidobacterium*		Increased	- Decreased total cholesterol concentrations - Reduced plasma triacylglycerol concentrations - Lowered Plasma CRP concentrations	[65]
		Firmicutes	*Lactobacillus*				
			*Enterococcus*				
			*Eubacterium*	*rectale*			
			*Clostridium*	*coccoides*			
		Firmicutes	*Clostridium*	*histolyticum*	Decreased		
	Dark chocolate made from Trinitario cocoa beans	Firmicutes	*Lactobacillus*		Increased	- Diminished Inflammatory Oxidative Damage (IOD) - Reduced LDL-Px - Increased lipoprotein O2 - Reduced corneocyte exfoliation	[82]
